# The Immunotherapy of Acute Myeloid Leukemia: A Clinical Point of View

**DOI:** 10.3390/cancers16132359

**Published:** 2024-06-27

**Authors:** Federico Mosna

**Affiliations:** Hematology and Bone Marrow Transplantation Unit (BMTU), Hospital of Bolzano (SABES–ASDAA), Teaching Hospital of Paracelsus Medical University (PMU), 39100 Bolzano, Italy; federico.mosna@sabes.it; Tel.: +39-0471-438807; Fax: +39-0471-438703

**Keywords:** immunotherapy, acute myeloid leukemia, bispecific antibodies, dual-affinity retargeting antibodies, chimeric antigen receptor cells, bioengineering, immune checkpoint inhibitors, T lymphocytes, NK cells, immune escape

## Abstract

**Simple Summary:**

Despite significant advancements, acute myeloid leukemia (AML) still remains characterized by a dismal prognosis in too many patients, and oftentimes, a cure can be achieved only after allogeneic hematopoietic stem cell transplantation (allo-HSCT), a procedure hampered by severe treatment-related complications. Despite this, new technologies have made it possible to harness the activity of the immune system against AML and use it as a new form of therapy, and intense research in the field has been made in recent years. This review aims to summarize the main concepts and strategies under study, considered from the point of view of the practicing hematologist, with special interest in the underlying biological principles and on treatment safety and efficacy. It will hopefully provide physicians, as well as the curious enthusiasts, with an updated, critically assessed description of immunotherapy as part of a more precise oncology approach to the treatment of AML.

**Abstract:**

The potential of the immune system to eradicate leukemic cells has been consistently demonstrated by the *Graft* vs. *Leukemia* effect occurring after allo-HSCT and in the context of donor leukocyte infusions. Various immunotherapeutic approaches, ranging from the use of antibodies, antibody–drug conjugates, bispecific T-cell engagers, chimeric antigen receptor (CAR) T-cells, and therapeutic infusions of NK cells, are thus currently being tested with promising, yet conflicting, results. This review will concentrate on various types of immunotherapies in preclinical and clinical development, from the point of view of a clinical hematologist. The most promising therapies for clinical translation are the use of bispecific T-cell engagers and CAR-T cells aimed at lineage-restricted antigens, where overall responses (ORR) ranging from 20 to 40% can be achieved in a small series of heavily pretreated patients affected by refractory or relapsing leukemia. Toxicity consists mainly in the occurrence of cytokine-release syndrome, which is mostly manageable with step-up dosing, the early use of cytokine-blocking agents and corticosteroids, and myelosuppression. Various cytokine-enhanced natural killer products are also being tested, mainly as allogeneic off-the-shelf therapies, with a good tolerability profile and promising results (ORR: 20–37.5% in small trials). The in vivo activation of T lymphocytes and NK cells via the inhibition of their immune checkpoints also yielded interesting, yet limited, results (ORR: 33–59%) but with an increased risk of severe Graft vs. Host disease in transplanted patients. Therefore, there are still several hurdles to overcome before the widespread clinical use of these novel compounds.

## 1. Introduction

Acute myeloid leukemia (AML) is the most common acute leukemia in adults. Following a clearer understanding of the pathogenesis of the disease achieved by recent advancements in flow cytometry and genetic sequencing, AML is currently thought to arise from the dysregulation of basic molecular mechanisms controlling hematopoietic differentiation and cellular proliferation [1,2,3,4]. This may be caused by either a massive event, such as one of the recurrent chromosomal translocations typical of the disease (e.g., t(8;21)(q22;q22), inv(16)(p13;q22)/t(16;16)(p13;q22), the alteration of the 11q23 locus, t(15,17)(q24;q21), t(9;22)(q34;q11)), or by the sequential acquisition of mutations in genes involved in epigenetic regulations (e.g., DNMT3A, TET2, IDH1, IDH2, ASXL1), cell differentiation (e.g., GATA2, RUNX1), nuclear transfer (e.g., NPM1), and the cell cycle, proliferation, and apoptosis (e.g., FLT3, N-RAS, K-RAS, KIT, TP53), often through a preleukemic myelodysplastic state. The updated 5th edition of the World Health Organization (WHO) classification of hematolymphoid tumors [3] and the International Consensus Classification of myeloid neoplasms and acute leukemia [4] both follow these acquisitions by defining disease categories based on genetic characteristics and pathogenetic features. Current AML therapy consists of a combination of cytotoxic chemotherapies (mainly based on an Anthracyclines + Cytarabine backbone) in younger patients and older “fit” patients with a low risk of severe (and potentially lethal) treatment-related complications, as well as the combination of hypomethylating agents (e.g., Azacitidine, Decitabine) together with the anti-bcl-2 drug Venetoclax in older or “unfit” patients [1,2,5,6]. At the same time, specific molecular therapies (e.g., FLT-3 and IDH1 inhibitors) have recently been added to this backbone in both settings and serve as examples of modern precision oncology [1,2]. More recently, Venetoclax has also been combined with intensive chemotherapy, in both upfront and rescue settings [7]. Nonetheless, despite all these advancements, the cure rate still rarely exceeds 60–70% in younger patients and is significantly lower at older age [1,2,8].

It is likely that additional improvement may be achieved by an immunotherapeutic approach. In fact, allogeneic hematopoietic stem cell transplantation (allo-HSCT) has consistently proven to be one of the most powerful strategies to achieve a cure, even though it is often at the cost of high treatment-related toxicity [9]. These results are the consequence of Graft vs. Leukemia effects, which have been consistently demonstrated starting from the pivotal study on identical twins vs. sibling donors [10] up to more recent studies [11], and are further demonstrated by the efficacy of donor lymphocyte infusions to eliminate residual disease after allo-HSCT [12]. Taken together, these studies provide proof-of-principle of the possibility to successfully harness the immune system against AML, especially in the context of low disease burden and good lymphocyte fitness [13] and after myeloablative conditioning [14]. A major point of interest in the immunotherapy of AML lies in the theoretical possibility to exploit the efficacy of immunosurveillance against AML without the hazards of allo-HSCT and the risk of Graft vs. Host disease (GVHD).

At the same time, the successful clinical development of monoclonal antibodies and antibody–drug conjugates (ADC) targeting tumor-related antigens has proved how immunological agents can directly target tumor cells with high specificity and efficacy.

Finally, in more recent years, bispecific T-cell engagers [15,16,17], chimeric antigen receptor T-cells (CAR-T) [18,19,20], and immune checkpoint inhibitors (ICPI) [21,22] have successfully entered the armamentarium against lymphoid malignancies. On the other hand, the clinical translation of new immunotherapeutic compounds in the therapy of AML is still at its dawn.

This review will summarize the principal approaches currently under research and the hurdles still to overcome in the immunotherapy of AML.

## 2. Identifying the Ideal Target

**Figure 1:**
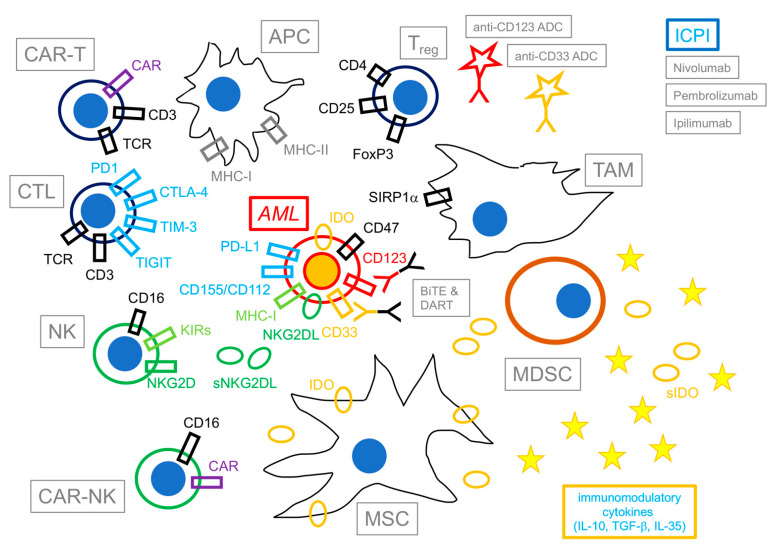
Immunotherapy of acute myeloid leukemia. This figure shows a schematic representation of selected components and agents involved in the immunotherapy of acute myeloid leukemia (AML). Cells exerting immune activity against AML are shown on the left of the figure; opposite to them, immunomodulatory cells and immunosuppressive agents are shown on the right. Potential immunotherapeutic drugs are shown on top of the figure. Besides exposing antigen-derived peptides from MHC to cytotoxic T lymphocytes (CTL, in black) and natural killer cells (NK, in green), AML blasts (in red) actively express immunomodulatory molecules (e.g., indoleamine 2,3 dioxygenase—IDO) and immune checkpoint ligands to evade immune reactions (e.g., PD-L1, CD155/CD112, NKG2DL), as well as maintain sensitivity to potential immunotherapeutic agents (e.g., antibody–drug conjugates—ADCs, bispecific T-cell engagers—BiTEs, and dual-affinity retargeting antibodies—DARTs) by the expression of lineage-restricted antigens (e.g., CD33, CD123). CTL/CAR-T and NK/CAR-NK cells, together with antigen-presenting cells (APCs) are fundamental in activating and eliciting the immune response against AML; opposite to them, T-regulatory cells (T_reg_), mesenchymal stromal cells (MSCs), myeloid-derived suppressor cells (MDSCs), and Ttmor-associated macrophages (TAMs) modulate the immune response by means of contact-dependent as well as soluble factors (represented as yellow circles—soluble IDO (sIDO)—and starlets—immunomodulatory cytokines e.g., IL-10, TGF-β, IL-35). Following therapy, immunotoxins and immune checkpoint inhibitors (ICPI) also contribute to the formation of the milieu.

Figure 1 summarizes the principal cells and factors involved in the immune reaction against AML in the bone marrow (BM) tumor microenvironment (TME).

Ideally, a perfect antigenic target for immunotherapy would involve high and homogeneous tumor-specific expression, low or absent expression by non-tumor cells, redundancy in its biological function (in order to reduce toxicity), cell surface expression, and high immunogenicity. Further complicating the issue, AML is a highly heterogeneous disease, with complex clonal composition [23] and the propensity to phenotypically change over clinical history [24]. As such, no ideal target has yet been identified for the immunotherapy of AML.

A selection of potential candidates is listed in Table 1. They may be conceptually divided as follows: **a.** lineage-restricted antigens (LRAs), characterized by widespread expression by both leukemic and other cell types, especially myeloid progenitors and the monocyte/macrophage system (e.g., CD33, CD123, CLL-1); **b.** leukemia-associated antigens (LAAs), being hyperexpressed by leukemic cells but also present in other cell types (e.g., FLT3, WT1); **c.** leukemia-specific antigens (LSAs), highly specific to AML cells and often produced as the result of a pathogenic mutation involved in leukemic development (e.g., mutated NPM1, mutated FLT3).

LRAs present advantages for clinical development because of their almost universal expression by AML cells, limited intrinsic variability, and easy accessibility on the cell surface. Therefore, despite a small therapeutic window for on-target, off-tumor effects and the possibility of the diversion of immune response by the interference of non-leukemic cells bearing the same antigens (a phenomenon known as “antigen sink”), LRAs are currently more advanced in clinical translation than LAAs and LSAs.

## 3. Monoclonal Antibodies, Antibody–Drug Conjugates, and Bispecific T-Cell Engagers

### 3.1. Monoclonal Antibodies and Antibody–Drug Conjugates (ADC)

Clinical results with unconjugated monoclonal antibodies have universally been dismal because of the many issues involving escape and resistance [53]. A notable exception may be the TIM-3 inhibitor Sabatolimab, currently being studied in a phase Ib/II trial (NCT04623216), alone or in combination with Azacitidine, for patients still with measurable residual disease (MRD^pos^ AML) after allo-HSCT. In the most recent report, 21 patients had been enrolled [54]. One DLT (a grade 3 myocarditis) was observed and recovered at drug suspension. Two cases of grade ≥ 3 neutropenia and one case of grade ≥ 3 thrombocytopenia were also noted. Five deaths occurred, all due to AML progression. Overall, tolerance was good, with no case of GVHD or other immune-related treatment-emergent adverse events (TEAE). A total of 30% of patients were still in remission after one year of treatment [54].

Better results have been obtained by using ADCs. One such instance is Gemtuzumab Ozogamicin, a rediscovered drug now part of standard therapy for chemosensitive AML, alone or in combination with a “3 + 7” chemotherapeutic backbone [26].

More recent ADCs include the following:

- INA03, an anti-CD71 (i.e., the transferrin receptor) ADC composed of a humanized monoclonal IgG4 and monomethyl-auristatin-E. A phase I/II trial on 20 relapsed and/or refractory (R/R) AML/ALL patients (NCT03957915) showed no DLT up to the highest dose of 2 mg/Kg, no grade 2–4 TEAE, and blasts reduction by >50% in 3 of 18 evaluable patients, at doses of >1 mg/Kg [55,56].
- AZD9829, an anti-CD123 antibody (Ab) conjugated to a topoisomerase-I inhibitor. It proved effective in vitro and in vivo on CD123-positive AML cell lines and BM-resident AML cells, with mild myelosuppression; it also showed 100% tumor inhibition in 13 AML patient-derived xenografts (PDX) regardless of the intensity of CD123 expression. It was administered with two weekly doses at 5 mg/Kg, it achieved >50% durable blast reduction in the blood of 7/13 (53.8%) and in the BM of 5/13 (38.5%) treated patients [57].
- Vobramitamab Duocarmazine also showed potent preclinical cytotoxic effects in vitro against CD276-expressing AML cells, even if no data from clinical studies are available yet. CD276 (B7-H3) is a common immune checkpoint inhibitor expressed by many hematologic malignancies, although not by HSC. Its expression was higher in AML cells of pediatric patients clinically considered to have poor prognosis because of the presence of KMT2A fusions (43.7%), KAT6A-CREBBP (91.6%), and CBFA2T3-GLIS2 (56.4%), as well as in AML cells from adult patients (58%). In both cases, CD276 expression correlated with inferior survival (5-yr EFS 35% vs. 44%) [48].
- Mesothelin, a novel LAA in AML, was expressed by 36% of patients in a recent series but not expressed by healthy myeloid cells. It is associated with promoter hypomethylation and has also been addressed as a potential target of ADC therapy by anetumab ravtansine [52].

### 3.2. Bispecific Immune Cell Engagers

More recently, the original concept of blinatumomab [15], a bispecific CD3xCD19 antibody favoring the formation of an immune synapse between acute lymphoblastic leukemia (ALL) blasts and T lymphocytes, has been used to develop bispecific agents for the immunotherapy of lymphomas [16,17] and AML. Different formats of bispecific compounds have been developed, ranging from bispecific antibodies/BiTE, half-life-extended bispecific antibodies/BiTE (which can be administered over longer intervals), and dual-affinity retargeting antibodies (DART) to more complex compounds, such as DuoBody antibodies, affinity-tailored adaptors modulating T lymphocyte function, and tetravalent bispecific antibodies [58]. A selection of these compounds currently in clinical trials is listed in Table 2.

Common problems in the clinical translation of these compounds are as follows:

- The existence of relevant on-target, off-tumor effects, determining myelotoxicity, and the occurrence of cytokine release syndrome (CRS), with a small therapeutic window [27,67]; differently from CD19 bispecific antibodies and CAR-T, though, immune-cell-associated neurotoxicity syndromes (ICANS) do not appear common with AML T-cell engagers (or even CAR-T) [27,63,67];
- The diversion of the T-cell engagers by the bulk of healthy cells expressing the same antigens (i.e., “antigen sink”) [27,67];
- The occurrence of T-cell exhaustion by chronic stimulation through continuous antigenic exposure within the myeloid compartment [68,69,70];
- The clonal selection of AML blasts expressing escape antigenic variants due to AML phenotypic heterogeneity, ultimately making AML eradication impossible;
- The clinical management of heavily pretreated, immunosuppressed patients affected by AML in rapid progression during the step-up phase needed to diminish the occurrence of severe CRS.

Despite these limitations, advanced clinical research is ongoing on many compounds, and especially on CD33, CD123 and CLL-1/CLEC12A.

A list of anti-CD33xCD3 T-cell engagers is as follows:

- AMG 330: this bispecific antibody was able to elicit the cytotoxicity of AML cell lines and patient-derived blasts and to sustain long-term T-cell stimulation in coculture experiments; in PDX, it managed to suppress AML primary xenografts across a wide range of effector–target (E:T) ratios [25]; in an ongoing phase I clinical trial (NCT02520427), it reached *complete remission or complete remission with incomplete hematologic recovery* (CR/CRi) in 7/60 treated patients (11.7%), with CR/CRi rates upward of 17% among patients treated with >120 mg/day as continuous IV infusion) [59]. The median duration of response was 58.5 days (range: 14–121). Although higher leukemic burden and lower AMG 330 exposure negatively correlated with outcome, there was no apparent correlation between clinical response and the expression level of CD33. The main toxicity was CRS, in 78% of patients (grade ≥ 3: 11%), and manageable pancytopenia.
- AMG 673: a CD33xCD3 bispecific antibody with extended half-life was developed in order to bypass a major disadvantage of bispecific engagers, that is, the need for continuous infusion due to short half-life. It has shown, so far, an overall response (ORR) rate of 18.5%, including 1 CRi, in an ongoing clinical trial on 27 evaluable patients, with an acceptable toxic profile (CRS rate 63% overall, grade ≥ 3: 18%) [60,61].
- AMV 564: another bivalent CD33xCD3 antibody, administered on 14 days during a 28-day cycle, obtained one CR, one Cri, and one PR among 35 evaluable patients, with anemia as the most common grade ≥ 3 TEAE (11% of series) (NCT03144245) [62].

Besides CD33, another relevant LRA in advanced clinical study is CD123 (i.e., the IL-3 receptor α-chain):

- Flotetuzumab (MGD006) is a CD123xCD3 DART given through continuous IV infusion in a weekly lead-in step-up dose (up to 500 ng/Kg/day). It led to 5 CR/CRi responses among 27 patients and to CR/CRi in 4/13 patients (31%) with primary chemorefractory AML; on the other hand, none of the 11 patients with relapsed disease achieved CR/CRi [62]. CRS was the most common TEAE, with no reported grade ≥ 3 CRS by prophylaxis with dexamethasone, early Tocolizumab use, and temporary dose interruptions. In the subsequent phase II trial at the recommended dose (500 ng/Kg/day), ORR was 30% (with CR/CRi of 27%), and OS was 10.2 months (range: 1.87–27.27) in patients achieving CR/CRi [63], with 6- and 12-month OSs of 75% (95% CI: 0.45–1.05) and 50% (95%CI: 0.15–0.85), respectively. CRS was 50% (grade ≥ 3: 7%). In a related study, the authors identified an IFN-γ-related gene expression and protein signature that managed to predict both chemotherapy resistance and response to Flotetuzumab, which was suggested as potential biomarker for patient selection [71,72].
- JNJ-63709178, another CD123xCD3 bispecific T-cell engager, has been tested on 62 R/R AML patients so far, with acceptable toxicity (CRS 44%, grade ≥ 3: 15%), but no data have been published yet on clinical results [64].
- Vibecotamab (XmAb 14045) is another CD123xCD3 bispecific antibody currently undergoing a phase I clinical trial. On R/R AML patients (*n* = 63 + 1 R/R B-ALL), it resulted in two CRs and one CRi when administered weekly at either 1.3 mg/Kg (the recommended dose) or 2.3 mg/Kg. Overall, CRS was 77% (grade ≥ 3: 11%) [65].

CD123 has been studied also in the setting of NK engagers:

- SAR443579 is a trifunctional CD123/NKp46/CD16 NK cell engager currently being tested in a phase I/II clinical trial [73,74,75]. In the most recent report, 59 adult patients across 11 dose levels (range: 0.01–6 mg/Kg/dose) had received treatment. A total of 18 patients (30.5%) had previously been transplanted, and 52 (88.1%) had prior exposure to Venetoclax. A median of 2 cycles, with a median treatment duration of 7.9 weeks (range: 1.0–66.0), has been administered. TEAEs were reported in 35 patients (59.3%), with grade ≥3 in 40 patients (67.8%). CRS was reported in 4 patients (6.8%), but no ICANS occurred. At the dose level of 1 mg/Kg/dose, 5/15 patients (33.3%) achieved CR/CRi, with a median duration of 48 weeks, and one patient was undergoing subsequent allo-HSCT. High variability in E:T ratios was noted, with the best response at 1 mg/Kg/dose. This is paired with preclinical studies showing bell-shaped dose-dependent anti-tumor activity in SCID mice intravenously injected with MOLM-13 AML cells [76].
- Another NK-cell engager, AFM28 (CD123/CD16A), has been successfully tested in PDX mouse models, with a dose-dependent control of tumor growth and improvement in the median life span of the mice by up to 66%. In an in vitro model of the BM niche (HOME), AFM28 in combination with allogeneic NK cells led to an effective reduction in CD123-positive leukemic blasts [77].

Other compounds under development include the following:

- MCLA-117, a CLEC12AxCD3 bispecific antibody: CLEC12A (i.e., CLL-1) has been found in the majority of AML cases, including their LSCs, but has not been detected in healthy HSCs [30,31,78]. Out of 26 evaluable patients, 4 showed ≥50% blast reduction (15.4%), with the main TEAEs being CRS (all grade: 32%; grade ≥ 3:2%) and hepatotoxicity (i.e., elevated liver enzymes, grade ≥ 3:8%) (NCT03038230) [66,79].
- XmAb18968 is a novel CD38xCD3 bispecific T-cell engager with a modified Fc domain. In a phase I study on R/R AML patients, 12 evaluable patients (31% previously allotransplanted, 92% with prior Venetoclax exposure) with high-risk genetic features (mutations of TP53: 31%; RUNX1: 23%; ASXL1: 15%) experienced meaningful responses in two cases that later proceeded to allo-HSCT as consolidation. No DLT, no grade ≥ 3 CRS, and no ICANS were observed [80].
- BOS-371 is an engineered humanized monoclonal antibody (mAb) targeting IL1 receptor accessory protein (IL1RAP). IL1RAP, expressed on the cell surface, is directly linked with FLT3 and c-KIT signaling and is overexpressed on LSC and myeloid progenitors in AML and high-risk MDS. Tested preclinically, BOS-371 has proven effective so far in coculture assays and in PDX mouse models of AML, where a significant in vivo decrease in disease burden (peripheral: >70%; BM: >50%) was observed [81].

Another therapy that appears to be conceptually relevant is T-cell engagers using LAA and LSA as targets, such as FLT3, WT1, and mutated NPM1:

- In the first case, AMG 427 is a CD3xFLT3 half-life-extended bispecific antibody tested in preclinical experiments, where it showed T-cell-mediated killing depending on FLT3 surface levels across high (>1:38) E:T ratios [35], and it is currently in a phase I trial (NCT03541369). Another CD3/FLT3 bispecific antibody, CLN-049, is also currently being tested [82].
- In the second case, an HLA-A2-WT1 CD3 T-cell engager recently failed to prove to be beneficial in a phase I/II trial on R/R AML patients [83], but many other compounds based on the same target are still being studied [84];
- Finally, NPM1 is currently being tested as a potential target in a variety of studies [41].

Even though clinical results are still limited, these data provide an overall interesting proof-of-concept on the safety and efficacy of this strategy, in line with previous experience with lymphoid malignancies [15,16,17]. Intriguingly, ICANS were absent in all these reports.

The main observed TEAE was CRS; in order to reduce this, as is common for bispecific antibodies for advanced lymphomas [16,17], a step-up approach has become standard in administering the compounds to allow higher doses, which appear necessary to overcome the “antigen sink” effect, often with steroidal prophylaxis and early Tocilizumab (an anti-ILR antagonist) as a treatment of CRS [59,62,63,65,79]. Even though a step-up approach has indeed reduced CRS occurrence and improved tolerability, it might have also partially reduced the efficacy of bispecific antibodies in heavily pretreated patients, who are prone to leukemia progression and infectious complications while undergoing the slow step-up phase.

A different approach to the problem may be the development of conditional bispecifics, activated in vivo only in the TME by the action of tissue proteases, as tested in some types of solid tumors [85,86].

A major problem of T-cell engagers, finally, is the lack of a sustained response following therapy, as a loss of efficacy and AML relapse are common over time, and long-term survival has been observed only in patients who have been further consolidated by allo-HSCT. This is a result of various causes, among which include the combined effect of the TME on immune cells activated by T-cell engagers and the occurrence of immune exhaustion over time.

In order to prevent this, following the experience with CD19/CD3 Blinatumomab [87], treatment-free intervals have been programmed during maintenance in the most recent trials. Furthermore, compounds such as FAP-4-1BBL (RG7826) and CD19-4-1BBL have been developed to be administered together with a bispecific antibody in order to provide a tumor-based costimulatory signal without systemic FcgR binding [88]. Finally, the use of checkpoint inhibitors together with bispecific antibodies (e.g., Pembrolizumab in combination with AMG33 in the clinical trial NCT04478695) is also being tested [89].

## 4. CAR-T Cells and Other Cellular Bioengineering

**Figure 2:**
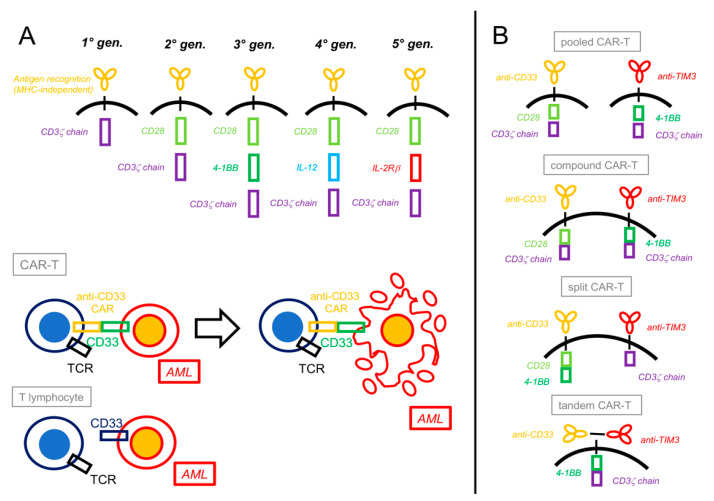
Chimeric antigen receptor T-cells constructs and platforms. (**A**) Chimeric antigen receptor (CAR) constructs have evolved over time from a basic form that only contained a CD3ζ chain intracellular signal domain (1st generation) to more complex structures that include one (2nd generation) or two (3rd generation) costimulatory domains (most commonly CD28 or 4-1BB). More recent CARs contain interleukin-producing sections (4th generation, e.g., IL-12) or intracellular domains of cytokine receptors (5th generation, e.g., IL-2Rβ). Besides acquiring the CAR, engineered T lymphocytes usually maintain their previous T-cell receptor (TCR), depending on the specific design. Upon recognition by their CAR, CAR-T cells activate against the target cells (e.g., CD33+ AML) by inducing their apoptosis. (**B**) “Dual CAR” platforms can generate different combinations of CAR-Ts: in the *pooled CAR-T*, two different clones, each with its specific CAR, are generated; in the *compound CAR-T*, both CARs, each complete with a costimulatory and an activating domain, are expressed by the same cell; in the *split CAR-T*, two different CARs (a chimeric antigen receptor and a chimeric costimulatory receptor) are present on the same cell and linked differently to activating or costimulatory domains; finally, in the *tandem CAR-T*, two antigen-recognizing CARs are both linked to the same costimulatory and activating domains.

CAR-T cells involve the use of bioengineered T lymphocytes endowed with the expression of a second *chimeric antigen receptor (CAR)* capable of recognizing a specific antigen in an MHC-independent manner, directly connected with a ζ-chain-stimulating domain and one (2nd generation) or two (3rd generation) costimulatory molecules (e.g., 4-1BB or CD28), and possibly more factors (4th and 5th generation, Figure 2) [18,90,91].

There are, at present, no commercially available CAR-T products for the therapy of AML. Despite this, significant research is being conducted, and several clinical trials are ongoing (Table 3). As per bispecific T-cell engagers, proof-of-principle has been demonstrated, even if clinical results are still limited, and issues with regard to target choice and toxicity are present. Myelotoxicity and potential long-term myeloablation due to the ablation of HSC as a result of targeting LRA and LAA is of special concern.

Early clinical studies with CAR-T cells for AML are currently ongoing:

- In one phase I/II trial (NCT03971799) on young R/R AML patients, autologous CD33 CAR-T cells were infused after lymphodepletion in 19 patients. The median time from enrollment to infusion was 47 days (24–242). Transient CD33 CAR-T expansion was detected in 9 subjects overall (47.4%) and in all 6 subjects at the highest dose level (100%). Transient myeloid aplasia occurred in 2 of 5 evaluable subjects (40%). One case of grade 4 DLT (i.e., a grade 4 CRS) was observed. CRS ≥3 occurred in 21% of patients [96].
- In another study (NCT02159495), CD123-specific CAR-T cells have been used against R/R AML (and BPDCN) with prior lymphodepletion and one or two CAR-T infusions [97]. Among seven patients (six post-transplant refractory AML; one BPDCN), one achieved second CR and proceeded to a second allo-HSCT, one remained in CR (acquired by prior bridge-therapy) and proceeded to allo-HSCT, and two more had significant blast reduction. Five of the seven patients (71.4%) had grade 1–2 CRS, and TEAEs were manageable. One patient with BPDCN achieved CR after a single dose of CD123-CAR-T, without CRS. No ICANS were reported [97].
- Another phase I dose-escalation trial is ongoing with CLEC12A/CLL-1 CAR-T cells in adult patients with R/R AML. In the latest report, 30 patients (mean prior therapy lines: 4; patients with prior allo-HSCT: 26.7%) had been treated, with a high CR/CRi rate (73.3%) but significant toxicity. In fact, all patients experienced CRS (grade 3–4: 12/30, 40%), myelosuppression was common (grade 3/4 neutropenia: 96.7%; anemia: 93.3%; thrombocytopenia: 100%), and one patient experienced grade 4 ICANS. When possible, cytopenia was successfully treated with haploidentical HSCT. Patients had a median PFS of 300 days and a median OS of 348 days [98].
- In a fourth phase I study (NCT03018405), twelve patients with various hematological malignancies (eight AML, three MM, and one MDS) received CYAD-01, a NKG2D-specific CAR product with broad specificity against a range of commonly expressed tumor antigens (MICA, MICB, and ULBP1-6). The CR/CRi rate was 42% (three of seven evaluable patients). CRS occurred in five of twelve patients (41.7%; grade 3: 16.7%). One patient, later consolidated with allo-HSCT, experienced long-term OS [99].
- Finally, CAR-T cells have been developed against C-C chemokine receptor 1 (CCR1, CD191), which is expressed on 75% of all AML samples and on HSC and T-cells at a minimal level. All preclinical studies were effective, with cytotoxicity in vitro and enhanced trafficking in the BM and spleen in vivo. Most interestingly, CAR-T expansion was seen in vivo even in the absence of a tumor, hinting at the possibility of activation also by bystander CCR1+ non-leukemic host cells. Signs of mild toxicity potentially caused by this on-target/off-tumor effect were observed. No clinical data are available yet [100].

In order to improve efficacy and reduce toxicity, dual-targeting CAR-T cells have also been developed. The rationale is to make T-cell activation dependent on two independent CARs, one linked to antigen recognition and connected with the ζ-chain domain and the other, named *chimeric costimulatory receptor (CCR)*, linked to a second signal activating the costimulatory molecule (Figure 2):

- CLL-1b/CD33b cCAR consists of two individually complete CARs linked by a self-cleaving peptide. As per the latest report, two advanced R/R AML patients have been tested (NCT03795779); both achieved CR and proceeded to allo-HSCT [101].
- In a different technical approach, a bispecific and split CAR-T (BissCAR) targeting CD13 and TIM-3 was developed through the use of nanobodies (heavy-chain-only antibodies with a small single variable domain) and proved effective in murine PDX models [46].
- Another dual CAR/CCR CAR-T (named ADCLEC.syn1), targeting ADGRE2 (with its CAR) and CLEC12A/CLL-1 (with its CCR), has been developed with the goal to target ADGRE2^low^ AML by the concomitant activation of both CARs while sparing ADGRE2^low^ normal HSC that lack CLL-1. The comparison between ADCLEC.syn1 and a conventional CAR-targeting CD33 in a MOLM13 PDX model has shown how ADCLEC.syn1 was superior in eliminating MOLM13 AML in the presence of bystander cells, thanks to its double CAR/CCR mechanism. In an R/R AML PDX model, both CAR cell types led to CAR-T cell in vivo activation and clonal expansion, but only ADCLEC.syn1 administration resulted in complete remissions; moreover, mice relapsing with AML after CD33-CAR-T treatment were still successfully treated by ADCLEC.syn1, achieving second remission. A clinical first-in-human trial is currently ongoing (NCT05748197) [102].
- A similar approach has been tested using CLL-1/CLEC12A and TIM-3 as target antigens for independent CAR and CCR. Preliminary results are encouraging; the potent inhibition (>95%) of leukemia blast viability and self-renewal was observed in coculture experiments. In vivo, these dual CAR/CCR cells prevented the engraftment of coinjected AML cells in PDX models and showed prolonged persistence up to 20 weeks after injection [103].
- Dual CARs targeting CD33 and TIM-3 were also recently combined by different methods and gating strategies. These were as follows: **a.** *pooled biclonal CAR+CAR*; **b.** *compound CAR/CAR*, with two independently complete and functional CARs; **c.** *split CAR/CCR*, where both stimuli needed to happen simultaneously on the CAR and CCR to activate the T-cells; and **d.** *tandem double-recognizing CARs*, with the two antigen-recognizing parts on the same construct [103] (Figure 2). In in vitro experiments with strong clinical implications, all four types of CAR-T cells showed better performance (i.e., higher avidity, enhanced cytotoxicity against AML, and a stronger production of proinflammatory cytokines such as IFN-γ and IL-2) as compared to single-targeting CAR-T cells. Important differences were noted, though: only *split CAR/CCR-T cells* did not show on-target, off-tumor toxicity against normal HSCs in colony-forming unit assays and thus appeared more specific; and *compound CAR-T cells* paradoxically expanded less and showed a higher expression of exhaustion markers in comparison to the other three CAR constructs [104].

Alternatively, in order to prevent excessive toxicity, the CAR construct can be engineered to modulate CAR-T cell activity in vivo by means of a second disposable signal, which is needed to activate the cells.

An example of this engineered construct is AVC-101 cells (i.e., UniCAR02-T-CD123), characterized by a universal CAR-T cell (UniCAR-T) modulated in vivo by a CD123-targeting module (TM) [105], which is administered as a continuous IV infusion. Due to the short half-life of the TM, it is possible then to modulate UniCAR-T activity by interrupting the administration. A recent phase I clinical trial of this strategy tested the TM in increasing doses, with UniCAR-T given just once, after standard lymphodepletion, at the start of TM administration. The induction cycle was planned as a 20-day continuous TM infusion, with the possibility of up to 3 consolidation cycles of 12 days each in responding patients. A total of 19 heavily pretreated patients (median: 4 prior lines; 12 patients with prior allo-HSCT) received the induction cycle, and 8 patients received at least one additional consolidation cycle. The ORR in the first 19 patients was 53% (8/15) for the R/R AML patients and 75% (3/4) for the MRD^pos^ population. CRS was observed in 12 patients (grade 3 CRS: 3), and 1 patient experienced grade 2 ICANS. all cases were resolved within 24 h of the interruption of TM administration. No treatment-induced lasting myelosuppression was observed [106].

- Another similar Fragment-antigen-binding (Fab)-based adapter CAR(AdCAR)-T cell platform has also been proposed, alternating between CD33 and CD123 as switchable targets [106]. In preclinical studies, T-cell exhaustion was prevented, and antigen heterogeneity was more efficiently dealt with by the possibility to modulate CAR-T cell activation by means of the infusion of different Fab-based adapters [107].

Finally, a novel and rather revolutionary concept to avoid prolonged myelotoxicity after CAR-T therapy against LRA shared by HSC and hematopoietic precursors involves the bioengineering of HSC to knock-out their CD33 expression prior to concomitant CD33 CAR-T infusion and allo-HSCT. This approach has proven feasible with the multilineage, long-term, hematopoietic recovery of CD33-KO HSCs in an in vivo model [108,109]. In a related study, three patients transplanted with CD33-KO HSC in an ongoing phase I/IIa trial (NCT04849910) displayed normal engraftment and could tolerate treatment with Gemtuzumab Ozogamicin after transplantation without cytopenia [110]. Despite this, the complexity of this HSC engineering will need a larger confirmation of many safety issues, including long-term engraftment stability and the safety of the gene-editing procedure, before translating from concept to a potential clinical strategy. Furthermore, it will have to comply with the same good manufacturing practice (GMP) standards as commercial CAR-T cells.

## 5. Natural Killer (NK) Cell-Based Immunotherapy

As T lymphocytes, natural killer (NK) cells infiltrate blastic BM, recognize AML blasts, and exert powerful cytotoxicity [111]. Compared to lymphocytes, they present quicker activation and generally shorter persistence. As lymphocytes, they are tightly regulated by a delicate dynamic balance between activating and inhibitory factors [112,113]. Among the activating triggers, the loss of MHC-class I by target cells, especially the HLA-A molecules, is one of the most relevant [111,114,115], and the loss of mismatched haplotype, killer immunoglobulin-like receptors (KIR) mismatch, and reduced NK alloreactivity have proven to be powerful prognostic indicators of patients experiencing AML relapse in the setting of haploidentical HSCT [116,117,118,119,120]. Furthermore, natural killer group 2D (NKG2D), NKG2C, and natural cytotoxicity receptors (NCR) act as activating triggers [112,121]; CD16 acts as FcgR of the IgG isotype, directing antibody-dependent cell cytotoxicity (ADCC); and KIRs in the HLA-B haplotype may exhibit stimulatory function, as well [114,122]. On the other hand, NK cells are negatively modulated by most other KIRs (KIR2DL 1/2/3/5, KIR3DL 1/2/3), checkpoint inhibitors (such as PD-1, CTLA-4, TIM-3, TIGIT), leukocyte immunoglobulin-like receptors (LIR), CD94/natural killer group 2A (NKG2A), Siglec-7/9, and CD200R [123].

A consistent amount of data points out how NK cells are dysfunctional in AML patients, as a consequence of AML activity and interactions in the TME [124,125,126,127,128,129]. As such, most attempts to use NK cells as immunotherapy have used allogeneic cells. Infusions of alloreactive NK cells have been used in several experimental studies, with conflicting, and mostly limited, results. In the setting of allo-HSCT, NK cell infusion after HLA-haploidentical HSCT appears well-tolerated and can consolidate engraftment through the depletion of recipient T-cells and antigen-presenting cells (APC) and by producing IL-10 [130,131]. In a phase I study, sequential doses of ex vivo-expanded cytokine-primed NK cells were beneficial in preventing leukemia relapse [132]; on the other hand, no benefit was observed by others [133], and results on leukemia control by the addition of NK cells to a standard conditioning regimen for allo-HSCT [134] and as a bridge to enable R/R AML patients to later undergo allo-HSCT [135] have also been disappointing.

Outside the field of allo-HSCT, NK cell infusions administered as rescue therapy in R/R AML patients similarly yielded limited results. The CR rate was 26.3% (5/19) in a historical study with haploidentical NK cells infused after high-dose Cyclophosphamide, Fludarabine, CD3 ex vivo depletion, and Il-2 administration (both ex vivo and in vivo) [136]. Later studies employing variations of this protocol have confirmed these results using more purified products (e.g., CD3-depleted, CD56-enriched haploidentical NK cells) and less immunosuppressive regimens (e.g., lower or no in vivo IL-2 to reduce infusion reactions). Overall response rates (ORR) still ranged from 20% to 37.5% in this series of 8–21 patients [137,138,139,140,141], and no randomized clinical study has been conducted as of yet.

When given as a maintenance method, NK cell transfer prolonged disease-free survival as compared to what was expected from historical data in two studies [139,140] but provided no advantage in another [142].

Several attempts are being made to improve these clinical results, as summarized in the following sections.

### 5.1. Combining Ab-Based Immunotherapy and NK Cells

The results of combined NK therapy with unconjugated antibodies have been generally poor [143,144,145].

### 5.2. Use of Bispecific NK-Engagers

The use of bispecific NK-engagers, such as SAR443579 [73,74] was discussed in the previous paragraph.

### 5.3. CAR-NK Cells

CAR constructs have been used to bioengineer NK cells, as was previously achieved with T lymphocytes:

- In the first-in-human trial of CD33-specific CAR-NK cells, 10 heavily pretreated patients with R/R AML were treated with anti-CD33 allogeneic CAR-NK cells derived from healthy donors. A total of 6/10 patients achieved MRD^neg^ CR at day 28, with favorable toxicity profiles (and only one grade 2 CRS) [146].
- NKX101 is an off-the-shelf CAR-NK cell population expanded from healthy donors and engineered to express a CAR-targeting NKG2D-L and a membrane-bound IL-15, the latter in an attempt to extend in vivo persistence and activity. It proved effective in eliminating target cell lines in preclinical experiments [147]. In a related clinical study, 6 patients with poor risk features were treated with multiple high-CAR-NK cell doses (1.5 billion each): 4/6 (66.6%) achieved CR/CRi, with 3 MRD^neg^ CR, and with 1 patient undergoing subsequent allo-HSCT. Ligand expression level was unrelated to response. NKX101 persisted for up to 3 weeks in pharmakokinetic testing despite being an allogeneic product. Myelosuppression and infections were the highest-grade adverse events. No cases of CRS, neurotoxicity, or GVHD were observed [148].
- CAR technology was also more recently used in combination with CRISPR/CAS9 gene editing to improve the efficacy of NK cells [149,150]. CAR33-NK cells were generated by the lentiviral transduction of NK cells from peripheral blood, CRISPR/CAS9 gene editing was used to knock out the killer cell lectin-like receptor C1 (KLRC1) gene, and cell expansion was conducted by an IL-15/IL-2-based medium. After gene editing, a 50% reduction in NKG2A cell surface expression was demonstrated on CD33-CAR-NK KO cells. These cells proved more efficient than regular CD33-CAR-NK cells in eliminating CD33+/HLA-E+ OCI-AML2 cells in xenograft models. No histologically detectable damage in the analysis of lung, liver, and colon was observed [149].

### 5.4. Checkpoint Inhibitors for NK Cells

IPH2101 and IPH2102 (Lirilumab) are antibodies targeting KIR2D, potentially preventing the interaction between KIR and HLA-C and enhancing NK cytotoxicity. When given as maintenance, though, Lirilumab did not perform more favorably than placebo in a phase II trial [151], nor in combination with Azacitidine in R/R AML patients [152]. Studies are ongoing with a combined LIR-1 and NKG2A blockade [153,154,155].

### 5.5. Allogeneic NK Cell Products

Intense research is currently concentrating on the development of an allogeneic “off-the-shelf” NK cell products. These would present several advantages, such as lower production cost, manufacturability at higher therapeutic dosages, the induction of a memory-like phenotype prior to use, appropriate KIR haplotype selection, cryopreservation and immediate application, and the possibility of additional genetic modifications [156].

Relevant recent experiences in this field are as follows:

- Allogeneic NK cells were administered in a phase I/II clinical trial with a dose-escalation design to 18 pediatric patients allocated in two groups (allo-NK vs. IL15-NK) according to KIR disparity between donors and recipients [157]. The most prevalent KIR-reactive clone was KIR2DL1 (55.6%), with a tendency to increase during the first year after transplantation. Severe acute GVHD (grade III–IV) manifested in 22.2% of patients, and severe chronic GVHD manifested in one patient (5.6%). The incidence of infection was 72% overall, and the incidence of vascular endothelial complications was 33%. One patient died because of relapse (5.6%), and four (22.2%) died because of transplant-related complications. One-year OS and DFS was 72.2%, with no statistical difference among the study arms [157].
- An NK cell line was generated from CD34+ HSC from *umbilical cord blood units* and used in a first-in-human trial that demonstrated NK cell expansion and maturation in vivo as well as a reduction in MRD without significant NK cell-related toxicity [158].
- CYNK-001 is an allogeneic NK cell population, enriched in memory-like CD56+CD3- NK cells, obtained from *placental CD34+ HSC cells*. In a phase I trial (NCT04310592), patients up to very old age (18–80 years) and affected by R/R AML (*n* = 28) or MRD^pos^ CR (*n* = 11) were treated with increasing doses. Two out of four patients at the highest dose level achieved a morphologic leukemia-free state on day 28, with one of them lasting up to day 120. One out of three MRD^pos^ CR patients achieved MRD negativity lasting up to day 120. Treatment was well-tolerated without DLT, even at the highest dose (1.8 billion cells × 4). No case of grade ≥ 3 CRS, ICANS (any grade), or GVHD was observed [159].
- Furthermore, another promising allogeneic NK cell product, *WU-NK-101* (i.e., *W-NK cells*) was recently developed. W-NKs express a memory-like phenotype as a result of cytokine programming during an expansion phase in coculture with IL-12/15/18. They show a specific transcriptome characterized by higher expression in genes related to metabolism, cell proliferation, and response to IL-15 activation; higher levels of activating receptors (i.e., 2B4, DNAM-1, NKG2D, NKp30), CXCR4 expression (important for BM homing); higher levels of cytotoxic effector proteins (granzyme B); and lower levels of inhibitory receptors [160,161,162]. Exposed to media from TME, their cytotoxic activity is more preserved than in naïve NK cells, as it is in in vitro hypoxic conditions mimicking the TME [161]. Finally, they discriminate cells from healthy human tissues, including normal blood cells. They are currently being evaluated in a phase I study on R/R AML patients (NCT 05470140), of which only early biological results have been published. In an analysis from 13 treated patients, W-NK cells showed high BM infiltration ability, lower gene signatures of T-cell exhaustion, higher cell cycling marker expression, and a more coordinated involvement of local immune cells in BM samples obtained from responders as compared to non-responders [163].
- Other studies are ongoing using human *NK cell lines*, such as NK-92 [164,165], or from *induced pluripotent stem cell (iPSC) lines* (NCT04023071) [166].

## 6. Immune Checkpoint Inhibitors (ICPI)

The pharmaceutical inhibition of checkpoint molecules has recently entered the armamentarium against Hodgkin’s as well as some non-Hodgkin’s lymphomas [21,22]. Several ICPIs have already been tested also in the case of AML, with somewhat disappointing results, and some concerns, especially in their use after allo-HSCT [167,168].

The combination of Nivolumab and Azacitidine, in fact, yielded a limited 22% of CR/CRi (ORR: 33%) in 70 treated R/R AML patients [169], with a median OS of 6.3 months overall and 10.5 months in patients treated as first-salvage therapy. The level of pre-treatment T-cell BM infiltration correlated with a higher response rate. The combination of both Nivolumab and Ipilimumab with Azacitidine, on the other hand, showed only slightly better results, with a CR/CRi of 36% and an ORR of 44% and grade 3/4 toxicity in 25% of patients, consisting of rash, pneumonitis, and colitis [170]. This may be the result of functional changes in checkpoint expression induced by IFN-γ, secreted by infiltrating T lymphocytes as part of a regulatory loop or by exposure to Azacitidine. In other studies, treatment with Azacitidine has shown a dose-dependent upregulation of PD-L1, PD-L2, PD-1, and, to a lesser extent, CTLA-4 in both AML blasts and T-cells, and it was linked to a shorter duration of response and a trend towards lower OS [171,172,173].

In another cohort, Pembrolizumab was combined with Azacitidine, with CR/CRi response in 14% and ORR in 18% of 29 R/R AML patients (median OS: 10.8 months) and CR/CRi 47% and ORR 59% (median OS: 13.1 months) in 17 newly diagnosed patients. Grade 3/4 immune-related adverse events were observed in 24% and 14% of patients of the two cohorts, respectively [174].

Durvalumab was also tested as a frontline therapy of AML in combination with Azacitidine in a randomized prospective setting against Azacitidine alone; there was no statistically significant differences in the ORR (31.3% vs. 35.4%) or CR rate (17.2% vs. 21.5%) between the two arms, with OS 13.0 vs. 14.4 months, respectively [175].

When combined with intensive chemotherapy, ICPIs improved results only slightly. Nivolumab was also added to intensive “3 + 7” Idarubicin/Cytarabine induction in a study on 42 AML patients [176]. ORR was 78% (CR 64%), with a median OS of 18.5 months for all patients and 18/34 patients (52.9%) undergoing allo-HSCT; 6/42 patients (14.3%) had grade 3/4 adverse reactions. In another study, Pembrolizumab was combined with high-dose Cytarabine in the treatment of 37 R/R AML patients. The ORR rate was 46% (CR/CRi: 38%), with a median OS of 8.9 months overall and 9/37 patients (24%) proceeding to allo-HSCT [177].

Caution has been expressed by the occurrence of severe (grade 3/4) or steroidal-refractory GVHD in patients treated with ICPI after (or soon before) allo-HSCT [167,168,178,179,180]. A therapeutic window of minimally 4 weeks has been proposed in the setting of lymphomas, although no evidence-based recommendation can be provided on this topic yet.

## 7. Vaccines

While a detailed analysis of attempts to vaccinate against AML is beyond the scope of this review (see Wu et al. for details) [181], it is relevant to mention the approach using dendritic-cell-based vaccines [182,183]. Among these, relevant results have been demonstrated with Vididencel, a vaccine obtained from a patient-derived AML cell line expressing WT1, PRAME, and RHAMM [183]. The ADVANCE II study is currently testing its use as a maintenance therapy in intermediate- and high-risk AML patients. Overall, 55 patients have been vaccinated so far, with a strong safety profile. T-cell responses against the antigens expressed by the vaccine have been proven through the analysis of blood samples and skin biopsies, and a reduction in relapse as compared to historical data has been observed [183].

## 8. Mechanisms of Immune Escape by AML

**Figure 3:**
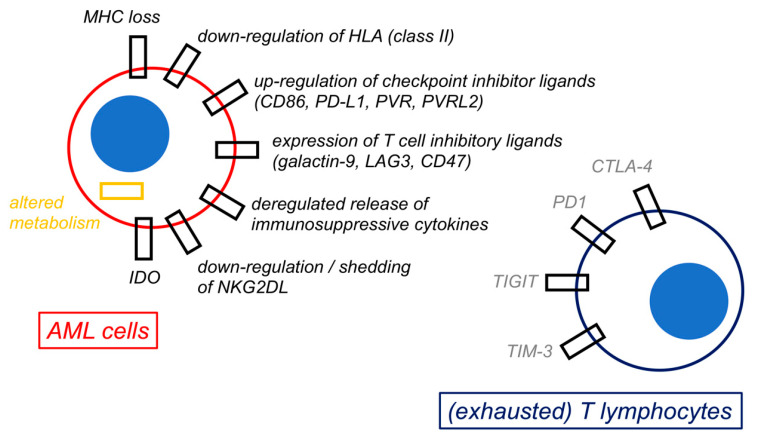
Mechanisms of immune escape by acute myeloid leukemia cells. **The figure** shows a schematic representation of selected possible mechanisms of immune escape by acute myeloid leukemia (AML) cells. All these biological changes by AML cells are ultimately responsible for immune evasion and for inducing an exhaustion phenotype in both T lymphocytes and natural killer (NK) cells (not represented).

A summary of the mechanisms of immune escape by AML is shown in Figure 3.

### 8.1. INTRINSIC Mechanisms by AML Cells

A primary mechanism of intrinsic resistance to immunosurveillance involves the loss of immunogenic stimuli, such as the mismatched HLA haplotype in the context of haploidentical allo-HSCT [116,117,118], as well as the downregulation of HLA class II molecules [184] and various LRAs (e.g., CD33, CD123, CLL-1) [25,27,28,30]. Strategies to restore HLA expression on AML blasts involve CIITA activation by IGN-γ [185,186], the activation of CtBP complex and FBXO11 expression [187], and, most interestingly, MDM2 inhibition [188].

On the other hand, AML hampers immune reaction through the acquisition of inhibitory checkpoint molecules as a consequence of stimulation by IFN-γ (produced by infiltrating T), by reactive oxygen species (mainly derived from macrophages) [89], or by proinflammatory cytokines [184]. The upregulation of PD-L1, PD-L2, CTLA-4, CD200, and CD47 in AML has been consistently observed [189,190] and negatively affects survival in animal models [191]. Furthermore, the downregulation of NKG2D by GATA2 overexpression is another common mechanism to evade immune surveillance [184] and is associated with an undifferentiated and poorly immunogenic stem-cell-like phenotype by AML cells [192].

Additional mechanisms of immune escape are constantly discovered and are likely to be highly individualized among patients. Among these, CD226 downregulation hampers NK alloreactivity, and CD38-directed CAR-NK cells may overcome this phenomenon [193]. Furthermore, STAT3-deficient AML cells evade NK cells in vitro by downregulating surface ICAM-1, and lower STAT3/ICAM-1 axis activity has been linked to poorer survival in patients [194].

### 8.2. Immune Exhaustion

Another important reason for clinical failure in immunotherapy is the occurrence of cell exhaustion following prolonged chronic antigen stimulation or CAR-T cell infusion. This is marked by the overexpression of CTLA-4, PD-1, LAG-3, TIM-3, and other checkpoint molecules on T lymphocytes and ultimately results in cell anergy instead of activation upon antigen recognition [184].

Interestingly, AML patients display a higher prevalence of T lymphocytes primed to exhaustion already at diagnosis. In a recent study, PD-1 expression was seen in 33.8% of peripheral T lymphocytes from patients with untreated de novo AML, and it was correlated with the expression of senescence markers, such as CD244 and CD57 [195]. This trend seems to increase over the history of the disease; patients experiencing relapse tend to have peripheral CD8+ T lymphocytes overexpressing PD-1 as compared to healthy donors [196] and higher PD-1 and OX40 expression by BM T lymphocytes as compared to diagnosis [197]. The same is true for NK cells [197], where contact-dependent TGF-β release by AML cells irreversibly damages NK cell reactivity through the signaling of the transcription factor BATF [198].

The occurrence of CRS after CAR-T therapy may also cause immune exhaustion [198]. The coincubation of CART-123 cells and cytokine-exposed primary AML blasts, mimicking in vitro AML cells primed by CRS, rapidly leads to functionally exhausted CAR-T cells displaying the characteristic PD1+/CD39+/CTLA4+/LAG3+ phenotype [199]. In this study, the phenomenon could be averted in vitro and in vivo by pharmacologically blocking JAK/STAT signaling.

Research from anti-CD19 CAR-T used in acute lymphoblastic leukemia has shown how the long-term persistence and activity of CAR-T clones are fundamental in determining long-term relapse-free survival [18]. Strategies to prolong CAR-T persistence have therefore been advocated for and involve primarily CAR engineering, such as by the introduction of double costimulatory or cytokine-producing domains (Figure 2). The dose and timing of the administration of CAR-T also appear to be pivotal in determining their in vivo persistence. Furthermore, the concomitant use of ICPI with this goal is currently under study. Caution needs to be applied, though, as the overstimulation of CAR-T cells by ICPI has been linked to increased CRS and GVHD [167,168]. In order to avoid that, strategies such as dual-gated CAR/CCR constructs [104] and the use of targeting modules [105,106] or Fab-based adapters (AdCAR) are being tested, with relevant results in preclinical studies [107].

### 8.3. Immune Modulation by the TME

Several components of the TME, as well as the cytokine milieu, are responsible for immune escape in AML (Figure 1). Among these, mesenchymal stromal cells (MSC) nurture and shield LSCs from immune surveillance in the hematopoietic niche [200,201,202] and can influence immune responses by direct contact and by the secretion of immunoactive molecules, among which is indoleamine 2,3 dioxygenase (IDO) [200,201]. This happens mostly upon activation by IFN-γ produced by infiltrating T lymphocytes [201,202] or following other types of conditioning. In fact, in a recent study, CAR-conditioned medium strongly activated NF-kB and STAT1 signaling in MCS, upregulating their secretion of several chemokines and immunomodulatory molecules (e.g., IL-6, IDO, PD-L1, CXCL10, CCL8, CXCL8, PTGES, and PTGS2) and actively suppressing CAR-T expansion in coculture experiments [203]. This effect was prevented by blocking NF-kB signaling in MSC. In addition, the supernatant from apoptotic MSC also reduced CAR-T expansion in vitro as well as in vivo, ultimately resulting in faster disease progression and reduced survival in a xenograft model [203].

Besides MSC, T_reg_ values are increased in the TME of AML and scarcely sensitive to chemotherapy, which, on the other hand, severely impairs cytotoxic T-cell responses over time; they are largely influenced by factors secreted by activated MSC and by AML cells themselves [201]. Furthermore, myeloid-derived suppressor cells (MDSC) also resist chemotherapy and are stimulated by inflammatory signals produced by infiltrating lymphocytes and macrophages [204]. They mostly act by releasing immunomodulative molecules, as part of a physiological feedback loop, and by restricting cellular trafficking and tissue infiltration by immune cells.

Finally, soluble factors in the TME (e.g., TGF-β, IL-4, IL-10 and soluble IDO) influence activation, proliferation, and killing by either T lymphocytes or NK cells (Figure 1). AML blasts may inhibit immune reaction by secreting IDO, Arginase-II, and prostaglandin E2 [184].

## 9. Conclusions

The proof-of-principle of the possibility to enhance immunosurveillance against AML has been proven by several compounds in preclinical and clinical studies. Among these, the most advanced options with regard to possible widespread use in patients appear to be T-cell engagers and CAR-T cells.

Step-up dosing has consistently ameliorated the safety and tolerability of T-cell engagers, even though results on patients with advanced R/R AML have so far been limited. The use of the same compounds earlier during the course of disease or in different settings (e.g., MRD^pos^ patients with minimal leukemic burden) might ameliorate results without renouncing good tolerability. At the same time, the common problem of production time and the other technical difficulties in manufacturing CAR-T for a large number of patients might be solved by using allogeneic “off the shelf” products, which, nonetheless, will still need very high production standards and clinical administration by highly trained clinical teams working under strict regulative standards. All these issues make T-cell engagers more likely to reach widespread clinical use in the treatment of AML before CAR-T. As is common in the present trials, the issue of excessive long-term myelotoxicity may be improved by subsequent allo-HSCT, a combined strategy in which immunotherapy might pose as a “bridge-to-transplantation” phase. For patients not undergoing allo-HSCT, the combination of immunotherapeutic compounds with drugs active on epigenetic regulation, such as, currently, hypomethylating agents (e.g., Azacitidine, Decitabine, Gaudecitabine), might provide synergistic clinical activity, as treatment with hypomethylating agents has been linked to antigen upregulation and increased immunogenicity by AML cells [205,206].

At the same time, there is a strong need to develop platforms that can reliably and consistently identify the patients who are most likely to respond to immunotherapy. Platforms identifying the genomic signatures of enhanced immune reactivity in AML patients, e.g., IFN-γ-related gene expression signatures [71], IED172 [207], or others [208], might help to sort out the best candidates. Major issues are still present in the definition of these genetic signatures, among which are their dynamic nature, with dramatic changes over the history of the disease, and problems of consistency and standardization among different laboratories; nevertheless, this seems like the path forward to produce real progress in the field. The application of new techniques, such as spatial proteogenomics, might also help in the understanding of the multifaceted interactions that exist in the AML TME. This is crucial in understanding how to avoid tolerance and enhance the effectiveness of immunotherapy.

As such, even if, at present, results may still be somehow disappointing for the practicing hematologist, we are only at the beginning of a new scientific journey that will most probably shape the future of AML therapy in the years to come.

## Figures and Tables

**Table 1:** Selected antigenic targets of immunotherapy in acute myeloid leukemia.

Antigen	Type	Expression on AML Blasts	Expression on LSC/HSC	Expression on BM Myeloid Precursors	Expression by Other Cell Types	Physiological Function	Immunotherapeutic Agents under Study	Clinically Approved Drugs	Ref.
CD33	LRA	+++ (100%)	+/+	+++	M/M system (Kupffer cells; microglia)	Sialic-acid-dependent cellular adhesion component	AMG330/AMG673 AMV-564/ADC/CAR-T	Gemtuzumab Ozogamicin	[25,26]
CD123	LRA	+++ (70–80%)	++/+	++	Gastrointestinal mucous membrane/bronchus	Interleukin-3 receptor	Flotetuzumab JNJ-63709178 Vibecotamab/CAR-T	Tagraxofusp (for BPDCN)	[27,28,29]
CLEC12A (CLL-1)	LRA	++ (80–90%)	+/−	−	Not reported	Immunomodulatory inhibitory C-type lectin-like receptor	MCLA-117 CAR-T	-	[30,31]
CD117 (c-kit)	LRA	++ (80–90%)	−/+	- (only in basophilic precursors)	Epithelial skin and breast cells; Cajal cells, melanocytes	KIT (stem cell/mast cell growth factor receptor)	CAR-T	TKI inhibitors (for Ph+ ALL or CML: Dasatinib, Ponatinib)	[32,33,34]
FLT3 (CD135)	LRA	++ (50–90%)	++/+	++	Gastrointestinal mucous membrane; central nervous system	Tyrosine kinase (cytokine receptor)	Bispecific antibody	TKI inhibitors (Midostaurin, Quizartinib, Gilteritinib)	[35,36,37,38]
IL1RAP	LRA	++ (80%)	+/−	++	Esophagus	IL1-receptor accessory protein	CAR-T	Anakinra	[39,40]
Mutated NPM1	LSA	+++ (30%)	++/+	++	Low tissue specificity	Nuclear chaperon	Preclinical	-	[41]
Mutated IDH1 (R132H)	LSA	++ (10–15%)	++/++	++	Low tissue specificity	Metabolic (Krebs cycle)	Preclinical	Ivosidenib	[42]
Lewis Y (CD174)	LAA	+	−/−	−	Gastrointestinal mucous membrane; epithelial cells	Unknown (carbohydrate blood antigen)	CAR-T	-	[43]
MUC1	LAA	++ (monocytic AML)	+/−	+	Gastrointestinal mucous membrane; epithelial cells	Mucosal protection	CAR-T	-	[44]
CD44v6	LAA	++ (60–70%)	+/−	+	Keratinocytes	Cellular adhesion component	CAR-T	-	[45]
CD244/ 2B4	LAA	++	++/++	++	M/M system	Activating/inhibitory receptor of NK cells	ICPI	-	[27]
TIM-3	LAA	++ (>80%)	++/-	++	M/M system	Immunoinhibitory co-receptor	ICPI/Antibody (Sabatolimab)	-	[46,47]
CD276 (B7-H3)	LAA	++	+/-	++	Low tissue specificity	Cell trafficking/immune checkpoint inhibitor	ADC (Vobramitamab Duocarmazine)	-	[48]
WT1 (Wilms’ tumor gene 1)	LAA	+++ (80–100%)	+/+	+	M/M system, spleen, kidney, heart, lung, prostate	Transcription factor	Vaccine	-	[49]
PRAME	LAA	++ (50%)	+/−	−	Testis	Unknown	Vaccine	-	[50]
RHAMM	LAA	+	−/+	+	Colon	Cellular adhesion component	Vaccine	-	[51]
Mesothelin	LAA	+	−/−	−	Mesothelia	Unknown (possibly cell adhesion)	ADC	-	[52]

ICPI: immune checkpoint inhibitor; CAR-T: chimeric antigen receptor T; AML: acute myeloid leukemia; LSC: leukemia stem cells; HSC: hematopoietic stem cells; M/M system: monocyte/macrophage system; BM: bone marrow; BPDCN: blastic plasmacytoid dendritic cell neoplasm; Ph+ ALL: Philadelphia-positive acute lymphoblastic leukemia; CML: chronic myeloid leukemia; LRA: lineage-restricted antigen; LAA: leukemia-associated antigen; LSA: leukemia-specific antigen.

**Table 2:** Selected bispecific T-cell engagers in acute myeloid leukemia trials.

Target	Drug Name	Type of Molecule	Clinicaltrials.gov Identifier	Clinical Phase	Indication	Primary End Points	Sponsor	Ref.
CD33	AMG 330	Bispecific antibody	NCT02520427	I	R/R AML	DLT, TEAE	Amgen	[59]
	AMG 673	Bispecific antibody	NCT03224819	I	R/R AML	DLT, TEAE	Amgen	[60,61]
	GEM 333	Bispecific antibody	NCT03516760	I	R/R AML	MTD, DLT, TEAE	GEMoab Monoclonals	-
	AMV-564	Tandem diabody	NCT03144245	I	R/R AML	DLT, TEAE	Amphivena	[62]
CD123	Flotetuzumab (MGD006)	DART	NCT02152956	I	R/R AML, int-2/high-risk MDS	DLT	Macrogenics	[63]
	Flotetuzumab (MGD006)	DART	NCT04158739	I	R/R AML (children + AYA)	DLT, TEAE	Macrogenics	-
	JNJ-63709178	Bispecific antibody	NCT02715011	I	R/R AML	DLT, TEAE	Janssen R&D	[64]
	Vibecotamab (XmAb 14045)	Bispecific antibody	NCT02730312	I	R/R AML	MTD, TEAE	Xencor	[65]
CD135 (FLT3)	AMG 427	Bispecific antibody	NCT03541369	I	R/R AML	DLT, TEAE	Amgen	-
CLL-1	MCLA-117	Bispecific antibody	NCT03038230	I	R/R AML, newly diagnosed elderly AML	DLT, TEAE	Merus	[66]
CD38	XmAb 18968	Bispecific antibody	NCT05038644	I	R/R AML	DLT, TEAE	Med. College of Wisconsin	-

DART: dual-affinity retargeting antibodies; R/R: relapsed/refractory; AML: acute myeloid leukemia; MDS: myelodysplastic syndromes; AYA: adolescent and young adults; DLT: dose-limiting toxicities; MTD: maximal tolerated dose; TEAE: treatment emergent adverse events.

**Table 3:** Selected chimeric antigen receptor T-cell trials in acute myeloid leukemia.

Target	Drug Name	Type of Construct	Clinicaltrials.gov Identifier	Clinical Phase	Indication	Primary End Points	Sponsor	Ref.
CD33	CD33CART	CAR-T cells (autologous/allogeneic)	NCT03971799	I/II	R/R AML (children and AYA)	Phase 1: MTD Phase 2: RR	CIBMTR	[92]
CD33/CLL-1	CLL-CD33 cCAR T	Dual cCAR-T cells	NCT03795779	I	R/R high risk hematological malignancies	MTD	iCell Gene Therapeutics	-
CD123/CLL-1	CD123/CLL1 CAR-T	Dual CAR-T cells	NCT03631576	II/III	R/R AML	LFS + TEAE	Fujian Med University	-
CD123	UCART123v1.2	Allogeneic CAR-T cells	NCT03190278	I	R/R AML	MTD, TEAE	Cellectis S.A.	-
	CD123CAR-41BB-CD3zeta-EGFRt T-cells	CAR-T cells	NCT03114670	I	R/R AML (after allo-HSCT)	TEAE, CART persistence, RR, OS	Aff. Hospital Academy of Military Med. Sciences	-
	CD123CART	CAR-T cells	NCT02159495	I	CD123+ R/R AML and persistent/recurrent BPDCN	TEAE, RR, OS	City of Hope (CA)	-
	CD123 CAR-T	CAR-T cells	NCT04272125	I/II	R/R AML	TEAE, RR	Chongqing Precision Biotech Co. Ltd.	-
MUC1/CLL-1/CD33/CD38/CD56/CD123	CLL-1 CAR-T	Multiple gene-engineered T-cells	NCT03222674	I/II	R/R AML	TEAE, RR, OS	Guangzhou Women and Children’s Med. Center	[93]
NKG2D	NKR-2 cells	CAR-T cells	NCT03018405	I/II	Various cancers including R/R AML	MTD, TEAE	Celyad Oncology SA	[94]
CLL-1, CD33 and/or CD123	CAR gene-engineered T-cells	CAR-T cells	NCT04010877	I/II	R/R AML	MTD, TEAE, RR	Shenzhen Geno-Immune Med. Institute	-
CD44v6	MLM-CAR44.1 T-cells	CAR-T cells	NCT04097301	I/II	R/R AML, CD44v6+ myeloma	MTD, TEAE, no vector replication	AGC Biologics	[95]

CAR-T: chimeric antigen receptor T; R/R: relapsed/refractory; AML: acute myeloid leukemia; MDS: myelodysplastic syndromes; BPDCN: blastic plasmocytoide dendritic cell neoplasm; CIBMTR: Center for International Blood and Marrow Transplant Research; AYA: adolescent and young adults; MTD: maximal tolerated dose; TEAE: treatment emergent adverse events; RR: response rate; LFS: leukemia-free survival; OS: overall survival.
